# Behavioural and electrophysiological modulations of onset primacy in visual change detection

**DOI:** 10.3758/s13414-025-03142-2

**Published:** 2025-08-15

**Authors:** Zeguo Qiu, Benjamin G. Lowe, Yasmin Allen-Davidian, Naohide Yamamoto

**Affiliations:** 1 School of Psychology and Counselling, Queensland University of Technology (QUT), Brisbane, QLD Australia; 2 Centre for Vision and Eye Research, Queensland University of Technology (QUT), Brisbane, QLD Australia

**Keywords:** Attentional capture, Change blindness, EEG, ERP, P100, P300

## Abstract

Generally, newly appearing objects attract observers’ attention more effectively than other types of change in an environment. While there is consensus about the existence of this phenomenon, it has been debated whether the new objects attain their primacy in an endogenous or an exogenous fashion. To contribute to this debate, the current study measured participants’ behavioural performance in detecting object appearance (onset) and disappearance (offset) while recording their electroencephalography. Some participants were trained to give priority to detecting offsets, and their data were compared against those of neutral (i.e., untrained) participants. This comparison revealed that the difference in behavioural response times between onset and offset detection was reduced after training, reflecting the degree to which onsets had attentional advantage over offsets in each group of participants. At the same time, amplitudes of the P100 event-related potential component were more differentiated between onset and offset detection in the trained participants than in the neutral participants. Critically, the modulations of the response times and the P100 amplitudes were not attributed to stimulus-driven effects because all participants were exposed to the same set of stimuli when the post-training results were obtained. Thus, these findings offer evidence that the relative efficacy of object onset in visual change detection is not purely a bottom-up phenomenon but is instead modulated by top-down processes.

## Introduction

Sudden changes happening in the surrounding environment often attract our attention in a rapid manner. The abrupt appearance of new objects (onsets) and disappearance of old objects (offsets) are two types of changes that are detected quickly and accurately, as they are crucial for our daily activities such as walking and driving. From an evolutionary perspective, it is hypothesised that primates may have developed heightened sensitivity to onsets as a survival mechanism, enabling quicker reactions to potential threats or resources in their surrounds (Zhang et al., 2022). While detecting new objects is generally advantageous and thus evolutionarily adaptive, there are circumstances where detecting disappearing objects could be important, such as a lifeguard needing to spot a swimmer who goes under.

Numerous studies have provided evidence for the efficient detection of sudden stimulus onset. The advantage of onsets in change detection was initially established in visual search tasks in which sensory transients uniquely occurred in the location of change (Jonides & Yantis, 1988; Yantis & Jonides, 1984). In these tasks, typically, a to-be-detected target (e.g., a letter ‘P’) is either displayed suddenly at a previously blank location or revealed by having parts of an existing placeholder stimulus fade away (e.g., the letter P is shown by extinguishing two lower-right segments of a digital figure eight). Observers detect the same target more quickly when it abruptly appears as an onset than when it emerges from the placeholder. This finding was later extended to change blindness paradigms in which no unique transients were associated with changes (Brockmole & Henderson, 2005a, 2005b; Cole & Liversedge, 2006; Cole et al., 2004). For example, in Cole et al.’s (2004) tasks in which participants attempted to locate one change that was made to an otherwise unchanging array of objects, the entire array disappeared once and then reappeared with the change. Thus, multiple objects produced sensory transients at the time of reappearance. In these tasks, the addition of a new object (onset) was localised more accurately than the removal of an old object (offset) and the alteration to an existing object (luminance or colour change). Based on this broad applicability across experimental paradigms, the relative efficacy of onset detection is termed *onset primacy* (Cole et al., 2004; Donaldson & Yamamoto, 2012).

In a series of experiments, Theeuwes (1991) explored effects of the preparatory attentional focus on onset and offset detection. In this task, participants searched for a target letter amongst three other non-targets. In the attention-unfocused condition, after the onset of a display, participants saw a central arrow that reliably cued the location of the target. Afterwards, a line segment either appeared suddenly (onset condition) or disappeared from the previous screen frame (offset condition). Results showed that both onset and offset changes attracted participants’ attention, as evidenced by substantially longer reaction times to the target, when the line segment was presented near a non-target. However, when participants’ attention was cued to the target location before the display onset (i.e., the attention-focused condition), the subsequent line segment did not attract attention in either onset or offset scenarios. Therefore, it was concluded that onset and offset detection is not an automatic process. Rather, it is susceptible to participants’ voluntary control (Theeuwes, 1991).

The strong modulatory effects of attentional control on onset or offset detection have been studied rigorously and well established. Specifically, both onset and, in particular, offset changes have been found to capture attention only when participants were primed with the same type of changes before the changes took place (i.e., ‘consistent attentional set’; Atchley et al., 2000). These findings are in line with the idea that attentional capture in general is modulated by top-down control (Atchley et al., 2000; Bacon & Egeth, 1994; Folk et al., 1992; Leber & Egeth, 2006; Yantis & Hillstrom, 1994; but see also Gibson, 1996; Theeuwes, 1992, 2004). Furthermore, studies using more naturalistic paradigms such as visual search with real-life objects showed that newly appearing objects, especially those that appeared while observers’ eyes were fixated elsewhere, attracted more saccades and fixations than objects that disappeared during the visual search (Brockmole & Henderson, 2005a, 2005b).

While the existing literature suggests that onset primacy persists across experimental paradigms (Adams et al., 2023; Brockmole & Henderson, 2005a, 2005b; Cole et al., 2003; Donaldson & Yamamoto, 2012; Yantis & Jonides, 1984), how this attentional bias towards onset over offset changes occurs is less well understood. In fact, the debate concerning the mechanisms that underlie onset primacy is still ongoing to date (Boot et al., 2005; Cole et al., 2004; Davoli et al., 2007; Franconeri et al., 2005; Miller, 1989; Theeuwes, 1994; Yantis, 1993; for a recent review, see Luck et al., 2021). Specifically, it has been suggested that the low-level characteristics of sudden onsets are more stimulating to the brain, especially to its visual regions, than other types of changes (e.g., offsets), resulting in a more efficient sensory processing of newly appearing objects (Franconeri et al., 2005; Miller, 1989). Proponents of this view have challenged the top-down account of attentional capture by onsets via an argument that the advantage of onsets can be reduced or eliminated when stimuli are equated in sensory transients they produce (Franconeri et al., 2005; Hollingworth et al., 2010). These results led to an alternative idea that onset primacy occurs primarily in a stimulus-driven, bottom-up fashion. However, it is not clear whether this idea fully explains onset primacy because onsets show enhanced detectability in change blindness too, that is, even when they do not generate spatially localised visual transients (Brockmole & Henderson, 2005a, 2005b; Cole & Liversedge, 2006; Cole et al., 2004).

On the other hand, it has also been proposed that our attentional system may be set to detect new objects in its default mode (Davoli et al., 2007; Donaldson & Yamamoto, 2016). It is hypothesised that creating mental representations of newly appearing objects requires more cognitive resources than deleting old representations of objects (Yantis, 1993). Hence, our attention network may always be tuned to onset detection to compensate for the need for greater processing. It is then inferred that, in order to process other types of change like offsets of old objects, a top-down shift from this default mode is required, resulting in a less efficient response (e.g., slower response time; Donaldson & Yamamoto, 2016). Therefore, one effective way to examine the underlying mechanisms for onset primacy would be to compare the brain’s responses to onset and offset changes. Specifically, in this study, we manipulated the shift from the default mode by training participants to enhance their offset detection and observed how it modulated behavioural and electrophysiological measures of onset primacy. By doing so, we sought evidence that the relative advantage of onsets can be altered by endogenously changing how offsets guide participants’ attention. Such a finding would indicate that onset primacy is not fully driven by bottom-up sensory processes but under the influence of top-down cognitive processes.

Of relevance, the P300 is an electroencephalography (EEG) component that often occurs around 300 ms after the onset of a stimulus over parietal brain areas. The P300 has been suggested to correlate with a variety of cognitive processes including how much of cognitive resources is allocated to certain stimuli during tasks (Hopfinger & Mangun, 1998; Koivisto & Revonsuo, 2003). In our recent EEG study, we presented participants with pairs of photographs in which there was a change in the second image, as compared with the first one (Van Pelt et al., 2025). Specifically, in the second image, either a new object appeared or an old object disappeared, termed the onset and offset trials, respectively. Participants were asked to quickly and accurately identify the locations of the changes. We found that the P300 was enhanced in the onset trials relative to the offset trials, which was accompanied by faster detection of the onset changes. We interpreted the stronger P300 as indicating preferential allocation of processing resources towards detecting onsets.

While not discussed in the previous study (Van Pelt et al., 2025), the P100 is another important neural marker in attention studies. With an early onset between 70 and 150 ms post-stimulus, the P100 is suggested to be primarily involved in the sensory processing of visual stimuli, but it has also been found to be modulated by cognitive factors such as task-relevancy and attentional load (Di Russo et al., 2001; Gazzaley, 2011; Mangun, 1995; Martínez et al., 1999; Rutman et al., 2010). In investigating how onset primacy occurs, the P100 can play a central role because of its early latency and specificity. While the robust response of the P300 to onsets and offsets makes it useful for research on onset primacy, it is not always straightforward to interpret P300 results because this component appears relatively late, creating room for several different processes to affect its amplitude (Polich, 2007). On the other hand, the P100 occurs shortly after stimulus presentation, making it more suitable for examining effects that are attributed to stimulus characteristics themselves (such as whether they show onsets versus offsets; Di Russo et al., 2001; Hopfinger & Maxwell, 2005). However, in our previous study, the P100 was not formally examined due to its focus on the P300 – effects of onset versus offset changes on the P100 were smaller than those on the P300, and we deemed that the study was under-powered to reliably detect the P100 effects (Van Pelt et al., 2025). In the broader literature, while the P100 has been shown to be sensitive to both onset and offset changes when they are noticed in a reflexive manner (Hopfinger & Maxwell, 2005), there have been few reports on attentional modulation of the P100 during volitional detection of onsets and offsets as to-be-detected targets.

Therefore, to gain insights into the underlying mechanisms that produce the relative efficacy of onsets over offsets in visual change detection, the present study examined both the P100 and the P300 in response to onsets and offsets of real-life objects. We adapted the behavioural paradigm developed by Donaldson and Yamamoto (2016) in which participants were trained to prioritise detection of offsets over that of onsets, and combined it with EEG recordings. This also resulted in more than doubling the number of trials as compared with our previous EEG study (Van Pelt et al., 2025), which helped improve the signal-to-noise ratio of EEG data so that they would reveal modulations of not just the P300 but also the P100. We aimed to investigate whether and how P100 and P300 amplitudes would be modulated when participants exhibited different degrees of onset primacy (indexed by a behavioural measure, i.e., response time). If these components were EEG correlates of onset primacy, their amplitudes should change as participants became adept at detecting offsets. Such modulation of the amplitudes would offer evidence that some processes that give rise to onset primacy – in this case, those that determine how offsets guide observers’ attention – are not fully exogenous but under endogenous control because, as shown in detail below, participants with offset-prioritising training and those without it were exposed to the identical set of stimuli while critical EEG data were recorded. Therefore, any modulation of the amplitudes cannot be ascribed to low-level sensory differences in the stimuli. Rather, it should be interpreted as an indication that top-down changes in attentional priority are reflected in differential appearance of the P100 and/or the P300 components.

## Method

This experiment was approved by the Office of Research Ethics and Integrity of Queensland University of Technology (QUT). It was conducted in accordance with the National Statement on Ethical Conduct in Human Research (National Health and Medical Research Council, 2018).

### Participants

Forty-three QUT students volunteered for the experiment in exchange for partial course credit. According to their self-report, they had normal or corrected-to-normal vision and no history of neurological disorders. All participants provided written informed consent prior to commencing their participation. For two of the participants, demographic details were not recorded. The remaining 41 participants consisted of 30 women and 11 men aged 17–28 years (*M* = 19.85 years, *SD* = 2.68 years). Forty of them were right-handed.

The sample size was set with the goal of having 15–20 participants per group (two groups in total – those who were trained to prioritise offset detection and those who did not receive this training) after excluding participants whose data were not usable for analysis (e.g., due to EEG recording artefacts). The target size of the subsample was determined by using sample sizes of two directly relevant previous studies as benchmarks. First, Van Pelt et al. (2025) found significant modulation of the P300 by the types of detected change (onset vs. offset) with 19 participants. Second, Donaldson and Yamamoto (2016) showed a reliable change in response times to onset and offset stimuli after participants had gone through the training. This study used 20 participants per group. These studies found large effects (*f* = 1.08 and 0.47), and thus we did not use them to derive the required (sub)sample size for the present study because it could be underestimated. For example, when the subsample size necessary for detecting the same training-induced alteration of response times was estimated via a priori power analysis using the effect size (*f* = 0.47), correlation between response times to onsets and offsets (*r* =.52), and sphericity parameter (ε = 1) observed in the Donaldson and Yamamoto (2016) study, G*Power (version 3.1.9.7; Faul et al., 2007) showed that it would be seven (with α =.05, 1 – β =.90, two groups, and two measurements per participant). Therefore, instead of aiming for such a small sample, we approximately replicated the sample sizes of the previous studies that employed the same EEG and behavioural paradigms. It should be noted that this made EEG analyses of the present study more powerful than those of the Van Pelt et al. study thanks to the substantial increase of the number of trials that went into the analyses.

### Stimuli and apparatus

**Fig. 1:**
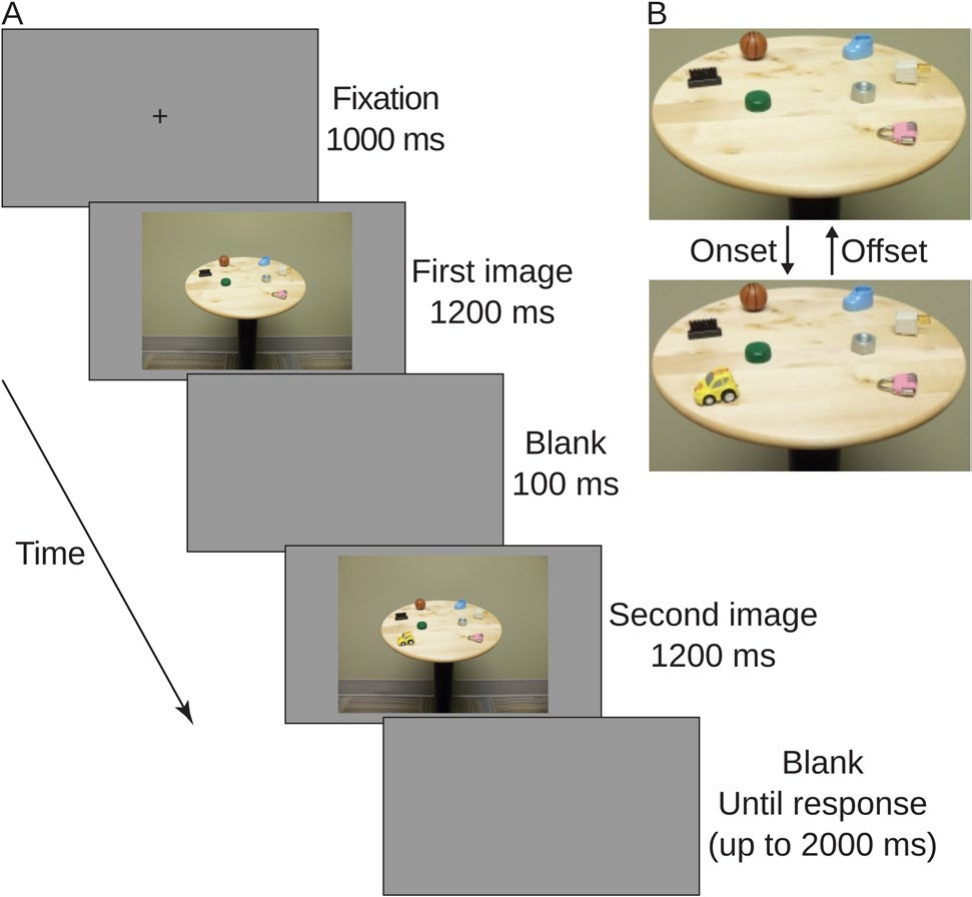
(**A**) The sequence of events that constituted a trial. This is an example of an onset trial, as a new object (the car) appears in the second image. (**B**) Close-up views of the tabletop and objects. The figure is adapted from Donaldson and Yamamoto (2012)

Stimuli of this experiment were identical to those used by Donaldson and Yamamoto (2016). They were coloured photographs that depicted a round tabletop (38 cm in diameter) on which six to nine objects were placed (Fig. 1). These objects were small toys and household items (approximately 4 × 3 × 2 cm of width, height, and depth). They were arranged in such a way that every object was visible in its entirety and the objects were distributed throughout the tabletop (e.g., when there were seven objects, three of them were on one side and four of them were on the other side). The photographs were shown in the centre of a 17-in. monitor with a resolution of 1,920 × 1,080 pixels. Participants were seated approximately 60 cm away from the monitor. At this viewing distance, each photograph subtended approximately 7° horizontally and 4° vertically. Stimulus presentation and behavioural data collection were managed by the PsychoPy software (Peirce, 2007).

EEG recordings were obtained using the BioSemi ActiveTwo 64-channel amplifier and the ActiView software (version 7.06) at a sampling rate of 1,024 Hz. Placement of the 64 EEG electrodes followed the International 10–10 system, with a common mode sense and driven right leg circuit as online recording references. No electrooculogram electrodes were used. Electrode offsets were kept within ± 25 mV before starting the experiment.

### Design and procedure

The current experiment employed a one-shot flicker paradigm (Cole et al., 2003), which was modelled after a typical change blindness paradigm that involves repetitive presentations of two different versions of the same scene (e.g., they differ by one object). The two versions are intervened by a short blank screen (thereby creating a ‘flicker’), removing local sensory transients that would otherwise be associated with the location of the difference. In the standard flicker paradigm, the two versions are repeatedly presented until the difference is detected. In the one-shot variant, participants make a response after viewing each version just once (Fig. 1A).

Participants were told in advance that they would view pairs of photographs that depicted multiple objects on a tabletop. They were informed that in each pair one object would either appear or disappear, and their task was to indicate as quickly and accurately as possible which side of the tabletop contained the change, regardless of whether it was appearance or disappearance of the object. Using a standard computer keyboard, they pressed the F and J keys for indicating the presence of a change on the left and right sides of the tabletop, respectively. Twenty-two participants began the experiment with these instructions (neutral group). The other 21 participants were additionally advised that appearing objects would generally allow for quicker and more accurate detection than disappearing objects, but they should pay closer attention to disappearing than appearing objects in this experiment (trained group). Participants were randomly assigned to the groups.

A pair of photographs displayed against a grey background formed a trial (Fig. 1A). The photographs were identical except that one of them had one extra object. Following a fixation cross that stayed in the centre of the screen for 1,000 ms, participants viewed the first image of the pair for 1,200 ms. This was followed by a brief blank grey screen (100 ms) and then the second image of the pair. Upon presentation of this image, participants were allowed to make a key-press response. The second image remained on the screen for up to 1,200 ms before it was replaced by another blank grey screen. Once the response was recorded or 3,200 ms passed since the appearance of the second image (whichever came sooner), the screen was cleared and the fixation cross appeared again to mark the beginning of the next trial. The time elapsed between the beginning of the second image and participants’ response was measured as a response time. Accuracy in indicating the location of a change was also recorded.

All participants went through three blocks of trials. First, they received a practice block in which they performed 16 trials. Stimuli used in this block did not appear again in the subsequent blocks. Participants were then given 320 trials in the training block, followed by another 320 trials in the testing block. In the neutral group, each of these blocks showed the same number of onset and offset trials – the practice block contained eight onset and eight offset trials; and the training and testing blocks were identical, showing 160 onset and 160 offset trials each that were created from the same set of stimuli. In the trained group, participants were exposed to more offset trials than onset trials prior to the testing block. These participants did 4 onset and 12 offset trials in the practice block, and 72 onset and 248 offset trials in the training block. Furthermore, the first 40 and last 40 trials of the training block were all offset trials so that they were primed with detecting offsets. Except for this manipulation, onset and offset trials were always presented randomly within each block of this experiment. Participants in the trained group then carried out the testing block, which displayed the same 160 onset and 160 offset trials as those used in the neutral group. Participants received no performance feedback throughout the experiment.

Unless noted otherwise below, each photograph pair was used to generate both onset and offset trials by reversing the order of presentation (Fig. 1B). This manipulation ensured that onset and offset trials were equated in low-level pictorial properties (e.g., colour, luminance, and contrast), object locations, and possible higher-order information (e.g., certain object configurations might make changes easier or harder to detect). The only exception to this was that in the training block for the trained group, some pairs only produced offset trials because this block presented more offset trials than onset trials. Most of the pairs were composed of photographs that showed seven or eight objects in various configurations. These pairs formed standard experimental trials in which a seven-object image was followed by an eight-object image (an onset trial) or an eight-object image was followed by a seven-object image (an offset trial). Additionally, pairs that contained photographs of six to nine objects were prepared for creating non-standard filler trials in which an eight-object image was followed by a nine-object image (an onset trial) or a seven-object image was followed by a six-object image (an offset trial). These filler trials were randomly intermixed with the experimental trials because, without the filler trials, the first image of a pair would have unequivocally determined the type of an upcoming change. Of the 320 trials in a training or testing block, 64 trials were filter trials. In the training block for the trained group, they consisted of 16 onset and 48 offset trials. In the other blocks, they were evenly divided into 32 onset and 32 offset trials. In all blocks, each object was used the same number of times as a changing object, and its location (left or right) was counterbalanced across trials.

### EEG pre-processing

Data from five participants were excluded from all analyses due to EEG equipment failure and recording error during the experiment. EEG data from the remaining 38 participants were pre-processed with EEGLAB (Delorme & Makeig, 2004) and ERPLAB (Lopez-Calderon & Luck, 2014). Through visual inspection of waveforms, electrodes that produced sustained noise and artefacts for over one-third of the experiment were identified and interpolated. Signals were re-sampled to 512 Hz offline, high-pass filtered at 0.1 Hz, and cleaned by EEGLAB’s CleanLine plugin (https://github.com/sccn/cleanline) to remove line noise.^1^ All signals were then re-referenced to the average of all electrodes. EEG data were segmented into epochs with a time window of 700 ms from the onset of the second image, using a pre-stimulus baseline (−100 to 0 ms). Independent-component analysis was performed on the epoched data to identify and remove eye-blink and eye-movement components in the signals. After the eye-related components were removed, the epochs were visually inspected again and those containing other artefacts were detected and removed on a trial-by-trial basis. Four participants were excluded for further analyses at this stage due to the limited number of epochs surviving these pre-processing steps – specifically, fewer than 30 trials for a given condition of interest remained. Therefore, the final sample included data from 34 participants (20 for the neutral group and 14 for the trained group). In these 34 participants’ data, on average, 81% of trials were retained after pre-processing.

To identify the electrodes and time windows for the event-related potential (ERP) components of interest (P100 and P300), we took a data-driven approach by running a mass univariate analysis that consisted of two-tailed one-sample *t*-tests against zero performed at each electrode in each time point over signals averaged across experimental conditions (Groppe et al., 2011). The analysis was done on the grand-averaged data, and therefore it was agnostic as to whether epochs belonged to onset or offset conditions. To evaluate statistical significance, we defined the family of tests using all time points within an epoch (0–700 ms) across all electrodes, and performed a cluster-based permutation test with 2,000 permutations to correct for multiple comparisons, with a family-wise α level of .050. To form temporal and spatial clusters, each electrode was considered a part of a cluster if it showed *p* < .050 in the one-sample *t*-tests at consecutive time points and if the electrode and its three spatially neighbouring electrodes showed *p* < .050 at the same time point. Results showed that the P100 was significant over PO3/4, PO7/8, POz, O1/2, and Oz between 129 and 145 ms, and the P300 was significant over P1/2, P3/4, Pz, PO3/4, and POz between 300 and 400 ms. These electrodes and time windows align well with those used for analysing the P100 and P300 components in previous studies (e.g., Di Russo et al., 2001; Hopfinger & Maxwell, 2005; Koivisto & Revonsuo, 2003; Van Pelt et al., 2025). Signals were averaged across these electrodes and time points to derive mean amplitudes.

### Data analysis

It was anticipated that participants would locate the changes with high accuracy in all conditions (Donaldson & Yamamoto, 2012, 2016). Thus, the analysis of behavioural data focused on response times, which were analysed separately for training and testing blocks. Specifically, for each block, we conducted a 2 (change type: onset vs. offset) × 2 (group: neutral vs. trained) mixed analysis of variance (ANOVA) in which the change type was a within-participant factor and the group was a between-participant factor.

Consistent with the behavioural data analyses, the EEG data extracted for each of the two components of interest (P100 and P300) were analysed with 2 (change type) × 2 (group) mixed ANOVAs for the training and testing blocks.

For both behavioural and EEG data, our main focus was on differences within the testing block, which allowed for fair comparisons between onset and offset trials as well as between neutral and trained groups. That is, this block was identical in both groups, containing an equal number of onset and offset trials that were created from the same photograph pairs. In addition, we analysed results from the training block to behaviourally confirm that the manipulations induced intended training effects in the trained group, and also to observe how the P100 and P300 components were modulated while participants went through the training. However, outcomes from the training block analyses must be interpreted with caution because the two groups received different stimuli, and in the trained group, onset and offset trials were not equated in terms of their numbers and stimulus characteristics.

All statistical analyses reported below were performed in R (version 4.4.1) using correctly performed experimental trials only. It was predicted that trained participants would exhibit onset primacy to a lesser extent compared to neutral participants during the testing block, which would be reflected in the behavioural and EEG measures (e.g., reduced advantage of onset trials over offset trials in response times). It may be worth clarifying that the training method employed in the present study was not to eliminate or reverse onset primacy, but to attenuate it (Donaldson & Yamamoto, 2016). That is, it was expected that the trained participants would still detect onsets more efficiently than offsets, but they should show a smaller difference between onset and offset detection than the neutral participants. In statistical terms, the training-induced modulation should be captured by significant change type × group interactions in the ANOVAs.

## Results

### Behavioural results

Participants’ accuracy in indicating the locations of change was examined to identify possible outliers who performed the task substantially worse than the rest of the groups, but no such participants were found. As expected, accuracy was high across all conditions (Table 1). This result helped rule out the possibility that speed-accuracy trade-offs confounded any of the findings from response times reported below.

**Table 1:** Behavioural accuracy (%) and response times (ms) per group, block, and change type

	Group	Training block				Testing block			
		Onset		Offset		Onset		Offset	
		*M*	*SD*	*M*	*SD*	*M*	*SD*	*M*	*SD*
Accuracy	Neutral	95.90	5.01	96.60	4.85	97.30	2.72	96.33	2.92
	Trained	95.66	4.52	96.57	2.02	96.88	3.72	96.26	2.36
Response time	Neutral	537.27	86.78	621.08	72.71	469.01	75.77	576.83	66.08
	Trained	528.98	81.90	560.76	59.69	463.87	75.10	532.91	65.25

**Fig. 2:**
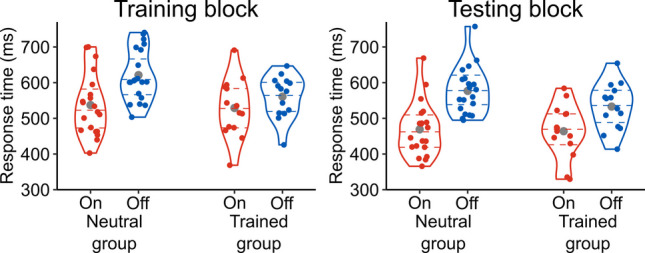
Distributions of individual participants’ mean response times per group, block, and change type. On = onset; Off = offset. Each coloured dot indicates the mean response time of one participant. The curves plot kernel density estimates. The dashed horizontal lines denote the first, second, and third quartiles. The grey dots show the mean response times collapsed over participants

**Fig. 3:**
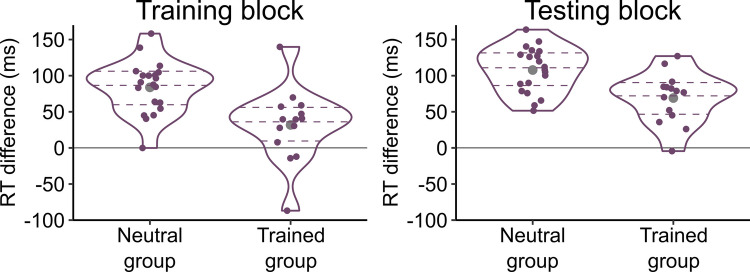
Differences in mean response times between onset and offset trials per group and block. RT = response time. Each coloured dot indicates the difference in mean response times between onset and offset trials within one participant. The difference was calculated by subtracting the mean response time in the onset trials from the mean response time in the offset trials. Thus, the positive values in the figure represent quicker responses to onsets than offsets (i.e., onset primacy). The curves plot kernel density estimates. The dashed horizontal lines denote the first, second, and third quartiles. The grey dots show the mean differences in response times between onset and offset trials collapsed over participants

To identify trials in which participants made responses too fast or too slow, means and standard deviations of response times were calculated per change type (onset and offset), group (neutral and trained), and block (training and testing), and trials in which response times were outside the *M* ± 3 *SD* range were considered to be outliers. This resulted in excluding 1.67% of correctly performed trials from the analysis. We then computed mean response times as shown in Table 1 and Fig. 2. In addition, to illustrate the degree of onset primacy at the level of individual participants, the difference in mean response times between onset and offset trials was calculated for each participant and plotted in Fig. 3. These data suggest that participants correctly responded to onset trials more quickly than offset trials, and the difference between the change types was smaller in the trained group than in the neutral group.

The above observations were confirmed by statistical analyses. For the training block, the ANOVA revealed a significant main effect of change type, *F*(1, 32) = 72.122, *p* < .001, η_G_^2^ = .149, and a significant interaction between change type and group, *F*(1, 32) = 12.148, *p* = .001, η_G_^2^ = .029. These results indicate that the training manipulation induced its intended effects such that onset primacy was diminished when the trained participants were instructed to prioritise detecting offsets and exposed to more offset trials than onset trials. The main effect of group was not significant, *F*(1, 32) = 1.784, *p* = .191, η_G_^2^ = .049.

The ANOVA for the testing block also revealed a significant main effect of change type, *F*(1, 32) = 265.361, *p* < .001, η_G_^2^ = .309, and a significant interaction between change type and group, *F*(1, 32) = 11.462, *p* = .002, η_G_^2^ = .019. As depicted in Fig. 3, the differences between onset and offset trials were greater in the neutral group (*M* = 107.82 ms, *SD* = 30.38 ms) than in the trained group (*M* = 69.04 ms, *SD* = 33.95 ms). These results indicate that the trained participants showed onset primacy to a lesser extent as compared with the neutral participants. The main effect of group was not significant, *F*(1, 32) = 1.045, *p* = .314, η_G_^2^ = .030.

### EEG results

#### P100

**Fig. 4:**
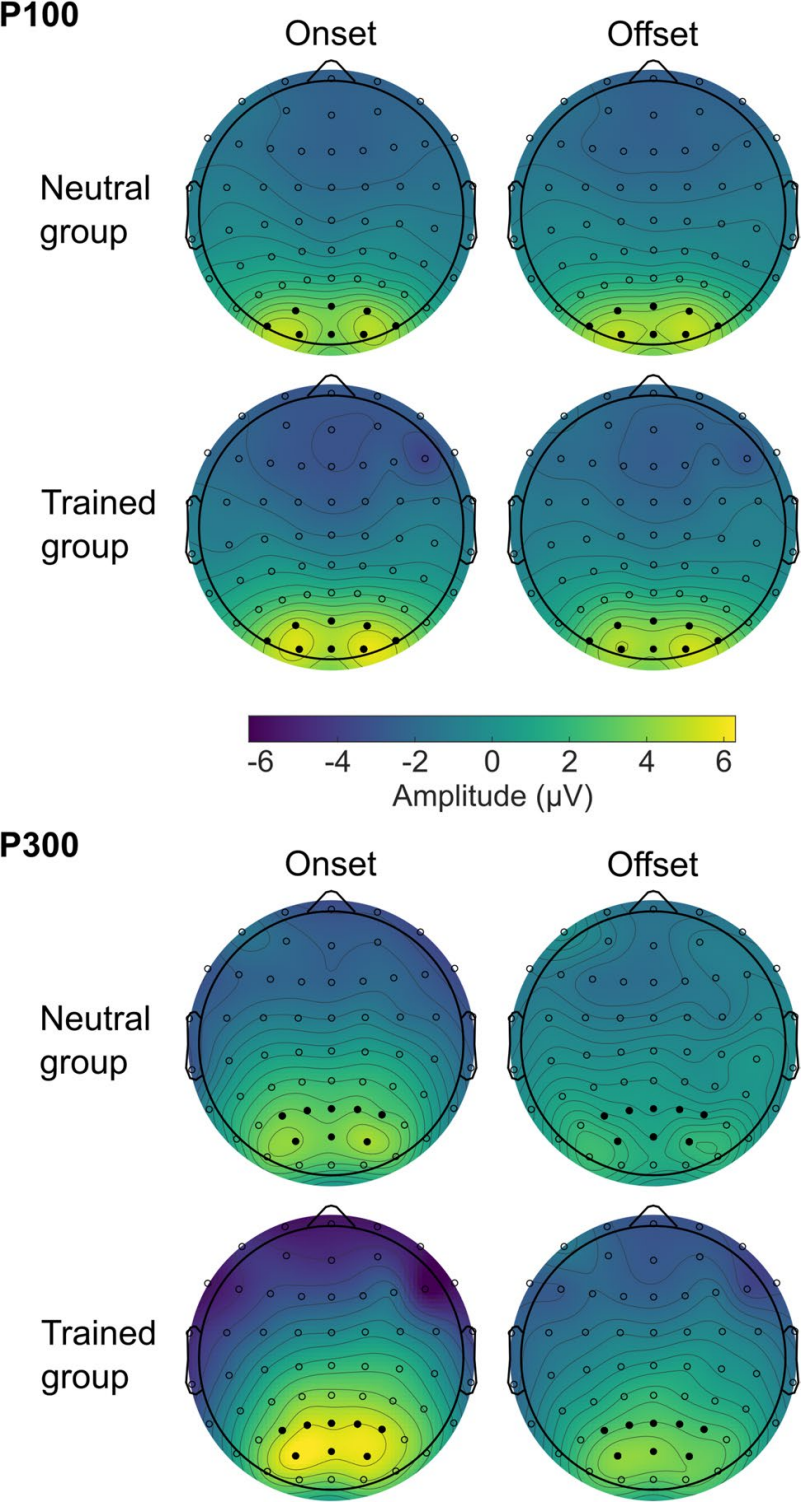
Topographical distributions of mean voltage in the testing block during the time windows of P100 and P300 components. Amplitude values were averaged across participants per group and change type in the periods of 129–145 ms (for the P100) and 300–400 ms (for the P300) after the appearance of a second image. Circles represent EEG electrodes. Filled circles indicate electrode clusters in which the P100 and P300 components were measured. They consisted of PO3/4, PO7/8, POz, O1/2, and Oz for the P100 and P1/2, P3/4, Pz, PO3/4, and POz for the P300

**Fig. 5:**
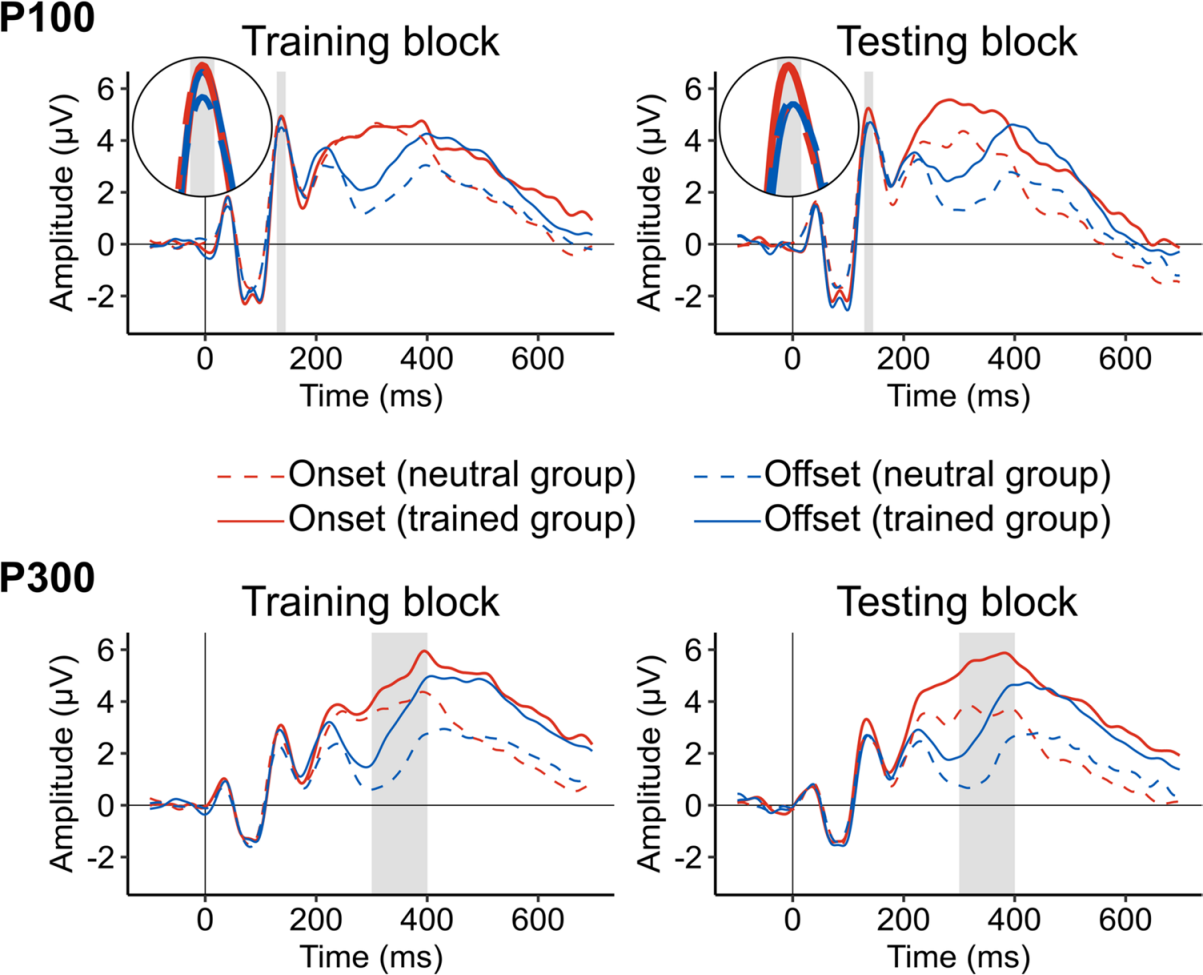
P100 and P300 grand-average time series per group, block, and change type derived from the following electrodes and time windows: PO3/4, PO7/8, POz, O1/2, and Oz between 129 and 145 ms (P100) and P1/2, P3/4, Pz, PO3/4, and POz between 300 and 400 ms (P300). Grey shading indicates these time windows. Circular insets in the upper panels show magnifications of the waveforms around the P100 time window. To plot the waveforms, band-pass (0.1–30 Hz) and notch (50 Hz) filters were applied to EEG data for smoothing purposes (see Footnote 1 for details)

**Fig. 6:**
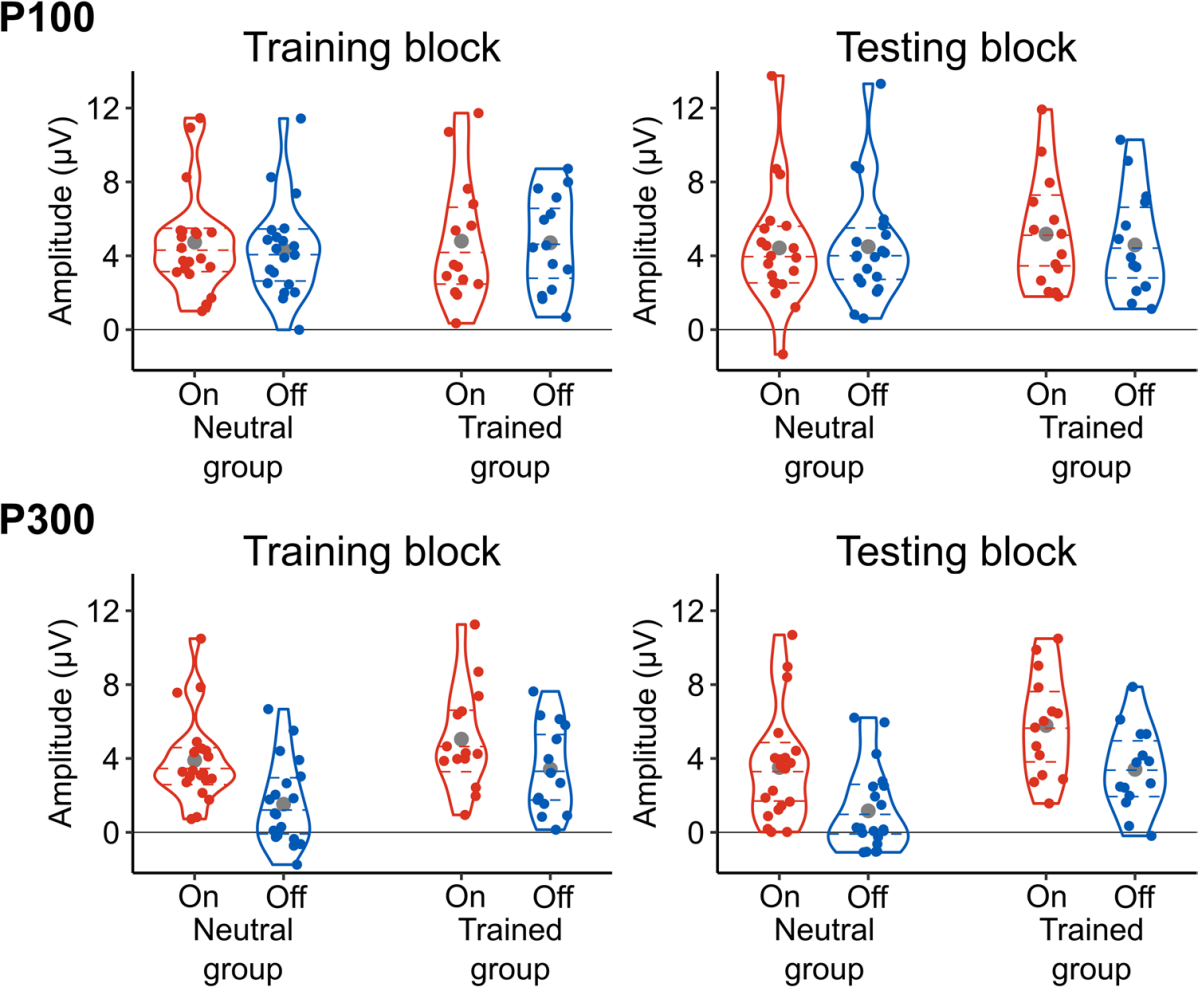
Distributions of individual participants’ mean P100 and P300 amplitudes per group, block, and change type. On = onset; Off = offset. Each coloured dot indicates the mean amplitude of one participant. The curves plot kernel density estimates. The dashed horizontal lines denote the first, second, and third quartiles. The grey dots show the mean amplitudes collapsed over participants

**Fig. 7:**
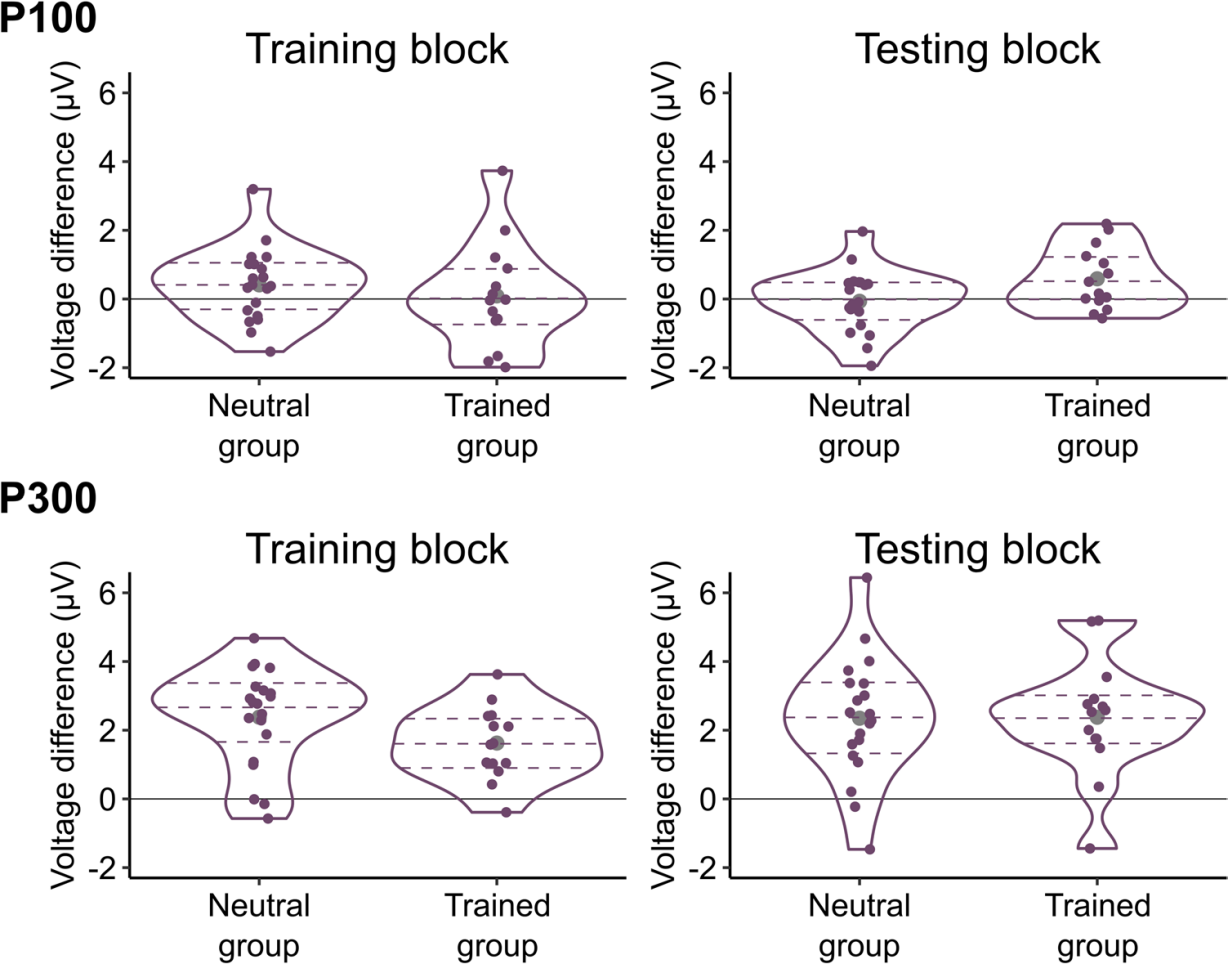
Differences in mean P100 and P300 amplitudes between onset and offset trials per group and block. Each coloured dot indicates the difference in mean amplitudes between onset and offset trials within one participant. The difference was calculated by subtracting the mean amplitude in the offset trials from the mean amplitude in the onset trials. Thus, the positive values in the figure represent greater amplitudes evoked by onsets than offsets. The curves plot kernel density estimates. The dashed horizontal lines denote the first, second, and third quartiles. The grey dots show the mean differences in amplitudes between onset and offset trials collapsed over participants

The scalp distributions of mean amplitudes in the P100 time window (129–145 ms) of the testing block are depicted in Fig. 4. Grand-averaged waveforms for the entire epoch (−100 to 700 ms) are plotted in Fig. 5. For both the testing block and the training block, mean amplitudes of the P100 component are shown in Table 2 and Fig. 6. The amplitude differences between onset and offset trials are illustrated at the level of individual participants in Fig. 7. They suggest that the P100 did not vary between conditions in the training block, but in the testing block it differed between the conditions such that the amplitude was modulated by the change types to different degrees in the neutral and trained groups.

**Table 2:** P100 and P300 amplitudes (μv) per group, block, and change type

	Group	Training block				Testing block			
		Onset		Offset		Onset		Offset	
		*M*	*SD*	*M*	*SD*	*M*	*SD*	*M*	*SD*
P100	Neutral	4.74	2.76	4.33	2.56	4.43	3.17	4.49	2.99
	Trained	4.80	3.38	4.71	2.62	5.17	3.06	4.59	2.88
P300	Neutral	3.90	2.38	1.52	2.25	3.51	2.99	1.16	2.25
	Trained	5.04	2.77	3.42	2.41	5.79	2.79	3.41	2.25

Consistent with the above observations, for the training block, the ANOVA yielded no significant effects, *F*(1, 32)s < 1.603, *p*s > .214, η_G_^2^s < .003. For the testing block, the ANOVA revealed a significant interaction between change type and group, *F*(1, 32) = 4.324, *p* = .046, η_G_^2^ = .003. As shown in Fig. 7, the amplitude differences between onset and offset trials were larger in the trained group (*M* = 0.59 μV, *SD* = 0.77 μV) than in the neutral group (*M* = −0.07 μV, *SD* = 0.77 μV). This result indicates that the degree of onset primacy as reflected in the P100 amplitudes was reliably different between neutral and trained participants. The main effect of change type and the main effect of group were not significant, *F*(1, 32) = 1.712, *p* = .200, η_G_^2^ = .001 and *F*(1, 32) = 0.162, *p* = .690, η_G_^2^ = .005, respectively.

#### P300

As in the P100 results, Table 2 and Figs. 4–7 display P300 data. They suggest that onset trials evoked larger P300 amplitudes than offset trials. In addition, overall, the P300 was greater in the trained group than in the neutral group.

For the training block, the ANOVA showed that the P300 was significantly larger in the onset trials (*M* = 4.37 µV, *SD* = 2.57 µV) than in the offset trials (*M* = 2.30 µV, *SD* = 2.47 µV), *F*(1, 32) = 86.220, *p* < .001, η_G_^2^= .161. The P300 was numerically larger in the trained group (*M* = 4.23 µV, *SD* = 2.54 µV) than in the neutral group (*M* = 2.71 µV, *SD* = 2.20 µV), but this difference did not reach statistical significance, *F*(1, 32) = 3.462, *p* = .072, η_G_^2^ = .091. The interaction between change type and group was not significant, *F*(1, 32) = 2.782, *p* = .105, η_G_^2^ = .006.

Similarly, in the testing block, the ANOVA revealed a significant main effect of change type such that the P300 was larger in the onset trials (*M* = 4.45 µV, *SD* = 3.08 µV) than in the offset trials (*M* = 2.09 µV, *SD* = 2.48 µV), *F*(1, 32) = 62.213, *p* < .001, η_G_^2^ = .180. The main effect of group was significant whereby the P300 was larger in the trained group (*M* = 4.60 µV, *SD* = 2.39 µV) than in the neutral group (*M* = 2.34 µV, *SD* = 2.49 µV), *F*(1, 32) = 7.012, *p* = .012, η_G_^2^ = .163. The interaction between change type and group was not significant, *F*(1, 32) = 0.002, *p* = .962, η_G_^2^ < .001.

Thus, the P300 results replicated the previous finding that greater amplitudes were evoked by onset trials than offset trials (Van Pelt et al., 2025). The effect of training was also visible in this component in that the trained group exhibited larger amplitudes than the neutral group. However, this effect was not specific to any one type of change.

## Discussion

The present study investigated whether onset primacy in visual change detection – the relative advantage of newly appearing objects in capturing attention – can be altered in a top-down fashion, and how this alteration is reflected in behavioural and electrophysiological measures. Some participants were first trained to prioritise detecting offsets and then performed a block of trials in which onsets and offsets were balanced in their frequency, location, and pictorial characteristics. Behavioural performance and EEG recordings of the trained participants were compared with those of untrained participants who went through the same testing block while being neutral in their explicit prioritisation of onset and offset detection (which means they would exhibit the standard onset primacy effects; Donaldson & Yamamoto, 2016). Given that the two groups of participants did not differ in the stimuli they viewed during the testing block, any behavioural and EEG differences observed between the groups would be interpreted to stem from top-down modulations of underlying processes. Results showed that the trained participants exhibited a lesser degree of onset primacy in response times, and the amplitudes of the P100 component were modulated correspondingly. In addition, the P300 component was stronger for onset changes than for offset changes, regardless of whether the participants were trained or not.

In the training block, the P100 did not differ between onset and offset conditions as well as between neutral and trained groups. It is hard to interpret these data because onset and offset trials were not equated in this block, and the two groups were exposed to different stimuli. Thus, it is possible that there truly were no effects of the conditions and groups on the P100, or there were effects but they were masked by the non-equivalence between the conditions and groups. On the other hand, results from the testing block, in which the conditions and groups were equated, allow for clear interpretations: There was evidence that early visual processes, as reflected by the P100, were affected by participants’ experience or prediction of the to-be-detected changes. Specifically, the P100 was more differentiated between onset and offset changes in the trained participants, which most likely resulted from increased exposure to offset changes and the instructions to give priority to them in this group. On the other hand, in the neutral group where such priming was not present, the P100 showed less difference between onset and offset trials.

One possible way of explaining these findings is that top-down instructions and frequent presentations of offset trials induced neuronal adaptation to offset changes in early visual neurons, and it helped increase the amplitude of the P100 during onset trials. The reasoning here is that processes underlying onset and offset detection are competing for common processing resources (Donaldson & Yamamoto, 2016; Van Pelt et al., 2025), and thus when one requires less of them due to adaptation (in this case, offset detection), the other (i.e., onset detection) can access more resources and boost its activity. Indeed, enhanced processing of primed stimuli in the brain is often represented by decreased, not increased, neuronal activation (e.g., Koutstaal et al., 2001). Importantly, this is not necessarily a stimulus-driven phenomenon: While earlier work suggests that the neuronal modulation is attributed to repetition of visual stimuli (Grill-Spector et al., 2006), more recent work emphasises the role of top-down processes such as predictions about the stimuli (Grotheer & Kovács, 2016). For example, after receiving many offset trials in the training block, the trained participants most likely expected more offset trials in the testing block because the instructions did not specify how frequently they would view onsets and offsets within each block. As a result, onset changes were counter to their expectation to some degree during the testing block, which elicited stronger early posterior ERPs (Baker et al., 2022).

One thing to note about the P100 results is that the effects of offset-prioritising training on the P100 were relatively small. This might have been a consequence of having fewer participants in the trained group (14 in this group vs. 20 in the neutral group) after dropping nine participants from analysis. We consider that the current results are reliable for the following reasons: First, they showed a specific pattern that was derived from our a priori hypothesis (i.e., the effect of change type was modulated by training). Second, the same results were obtained when raw EEG data were processed differently by filtering out high-frequency components that were shown to affect the P100 amplitude (Porcaro et al., 2011),^1^ indicating the robustness of the pattern exhibited by the P100. Taken together, we conclude that the observed patten did reflect a systematic modulation of the P100 amplitude caused by the training manipulation.

Unlike in the P100, effects of offset-prioritising training on the P300 were observed only across the groups, not specific to onset or offset detection. Interestingly, the trained participants, who exhibited onset primacy to a lesser extent, showed larger P300 amplitudes than the neutral participants. This suggests that the P300 does not simply reflect the amount of processing resources that are currently assigned to onset and offset detection (Van Pelt et al., 2025). Instead, it is possible that the P300 mainly represents the dynamic processes for moving resources between competing tasks, and once moved, actively maintaining them for the task to which they have been allocated. This view explains the trained group’s increased P300 amplitudes in both of the blocks because in the training block, the trained participants had to reallocate resources from onset detection to offset detection; and in the testing block, these participants attempted to maintain this allocation by resisting the intrinsic tendency of allocating the resources back to onset detection (Donaldson & Yamamoto, 2016). Neither of these processes were engaged in the neutral participants, who only needed to hold the resources that had been allotted to onset detection in the default mode. This still elicited the P300, but in smaller magnitude as compared with the trained group.

In addition to the effect of training, the P300 showed that its amplitude was greater across the board during onset detection than offset detection. This result, particularly as seen in the neutral group, is a direct replication of our previous finding (Van Pelt et al., 2025). It was not surprising to find the same pattern in the trained group because onset primacy was expected to sustain in this group – the training was meant to reduce the degree of onset primacy, not to eliminate it (Donaldson & Yamamoto, 2016). However, for this very reason, it was counter to our prediction that the amplitude difference between onset and offset trials was not modulated by the training. This insensitivity of the P300 leads to a conjecture that this component represents mechanisms that make onset primacy so robust: Onset primacy is ubiquitous in many different paradigms (Adams et al., 2023; Brockmole & Henderson, 2005a, 2005b; Cole et al., 2003; Donaldson & Yamamoto, 2012; Yantis & Jonides, 1984), and it is not easily malleable (Donaldson & Yamamoto, 2016). Considering that the enhancement of the P300 is an established neural marker of attentional allocation (e.g., Nash & Fernandez, 1996; Polich, 2007), one possibility is that the always high P300 amplitude in response to onset changes reflects oversupply of attention to newly appearing objects, which maximises the likelihood that they are successfully and swiftly detected. Indeed, as these objects could signal important sudden changes that require immediate action, it would make sense if our attentional system had a fail-safe set-up that could ensure proper prioritisation of onset detection under any circumstances. In line with this idea, studies on non-human primates have provided direct evidence for enhanced neuronal responses to onset stimuli in early and higher-order visual cortices (Luck et al., 1997) as well as in the fronto-parietal areas (Goldberg et al., 2006).

While the present study focused on the P100 and P300, there are other ERP components that are known to index attentional modulation. In particular, when targets in the left or right of eye fixation are attended, early posterior ERPs are elicited more prominently in one hemisphere than the other. Notable examples of such ERPs include the visual orienting activity (McDonald et al., 2022), N1pc (Wascher & Beste, 2010), and N2pc (Luck & Hillyard, 1994). This study was not suitable for observing these lateralised ERPs because participants’ fixation was not strictly controlled while they viewed stimuli. In the future, it would be useful to ensure that fixation is maintained in the centre of a display by either tracking eye movements or adding a vigilance task (e.g., the fixation cross occasionally changes its colour and participants must respond to it). This improvement would enable the current paradigm to investigate the attention-sensitive lateralised ERPs, thereby providing greater insights into neural and cognitive processes that underlie onset and offset detection.

Another important consideration for future studies is that modulation of onset primacy via training can be done in different ways. The current approach was to enhance offset detection by having participants carry out more offset trials than onset trials under the explicit instructions that they were to pay more attention to offsets than onsets. While this training was successful, the unbalanced presentation of onset and offset trials introduced two possible issues. First, the fact that these participants selected offsets more often than onsets as targets could affect their attentional priority by itself, independently of top-down and bottom-up factors – that is, the effect of selection history can be a third factor of attentional control, which has traditionally been studied under a dichotomous top-down versus bottom-up framework (Anderson et al., 2021; Awh et al., 2012). This issue could make it challenging to interpret the current findings in terms of whether they resulted from top-down or bottom-up processes. However, it is important to note that in this training method, just doing more offset trials is not sufficient to modulate onset primacy; rather, it has to be accompanied with the top-down instructions to give greater attention to offsets (Donaldson & Yamamoto, 2016). Thus, although the effect of selection history was not ruled out in this study, it was likely that top-down processes played a crucial role in shaping the current findings.

Second, the unbalanced presentation of onset and offset trials made this study unsuitable for directly comparing data between training and testing blocks because they showed different stimuli. This issue was particularly applicable to early ERPs including the P100 that tend to have increased sensitivity to low-level stimulus characteristics (Johannes et al., 1995; Luck & Hillyard, 1994). In the future, it would be important to manipulate onset primacy while keeping the blocks equivalent in the stimuli they use. Such a study will allow for fine-grained observations of how the measures of onset primacy are altered post training. For example, does the alteration occur after training because the P100 gets smaller in offset trials or larger in onset trials, or both? Strict comparisons between the blocks will provide unequivocal answers to these questions.

In conclusion, by examining behavioural and EEG responses to appearing and disappearing objects while holding stimuli constant across two group of participants, we found that these measures of onset primacy in change detection can be modulated in a top-down manner. Specifically, whether or not participants were trained to prioritise detecting offset changes altered the degree to which response times and P100 amplitudes were differentiated between onset and offset trials. These findings indicate that processes that underlie offset detection are permeable to top-down factors, offering evidence that onset primacy is not a fully automatic, bottom-up phenomenon. In addition, the P300 component was consistently stronger for trained participants and for onset changes, and these results provided additional insights into possible mechanisms that give rise to onset primacy. Some of these P300 results were not predicted a priori, however. Thus, it is important to follow them up via hypothesis-driven research in the future. This research can help understand why onset detection is so robustly prioritised in the attentional system.

## Data Availability

Data collected and analysed in this research are available via the Open Science Framework at https://osf.io/3b9yf/.

## Footnotes

1 EEG data were also processed in a conventional fashion by applying a band-pass filter (0.1–30 Hz) and using a notch filter (50 Hz) instead of the CleanLine plugin for line noise removal. This version of the EEG data, which is available at https://osf.io/3b9yf/, yielded the same P100 results as those reported in this article.

## Code availability

Custom code for EEG data processing is available via the Open Science Framework at https://osf.io/3b9yf/.

## Declarations

### Open practices statement

The availability of research materials is as declared above. The experiment was not pre-registered.
